# Isolation and Characterization of a Monobody with a Fibronectin Domain III Scaffold That Specifically Binds EphA2

**DOI:** 10.1371/journal.pone.0132976

**Published:** 2015-07-15

**Authors:** Seung-Hwan Park, Sukho Park, Dong-Yeon Kim, Ayoung Pyo, Richard H. Kimura, Ataya Sathirachinda, Hyon E. Choy, Jung-Joon Min, Sanjiv Sam Gambhir, Yeongjin Hong

**Affiliations:** 1 Department of Nuclear Medicine, Chonnam National University Medical School, Gwangju, Republic of Korea; 2 Department of Microbiology, Chonnam National University Medical School, Gwangju, Republic of Korea; 3 Molecular Imaging Program at Stanford, Department of Radiology, Bio-X Program, Stanford University, Palo Alto, CA, United States of America; University of Parma, ITALY

## Abstract

Monobodies are binding scaffold proteins originating from a human fibronectin domain III (Fn3) scaffold that can be easily engineered with specificity and affinity. Human EphA2 (hEphA2) is an early detection marker protein for various tumors including lung, breast, and colon cancer. In this study, we isolated two hEphA2-specific monobodies (E1 and E10) by screening a yeast surface display library. They showed the same amino acid sequence except in the DE loop and had high affinity (~2 nM Kd) against hEphA2. E1 bound only hEphA2 and mEphA2, although it bound hEphA2 with an affinity 2-fold higher than that of mEphA2. However, E10 also bound the mEphA6 and mEphA8 homologs as well as hEphA2 and mEphA2. Thus, E1 but not E10 was highly specific for hEphA2. E1 specifically bound human cells and xenograft tumor tissues expressing hEphA on the cell surface. *In vivo* optical imaging showed strong targeting of Cy5.5-labeled E1 to mouse tumor tissue induced by PC3 cells, a human prostate cancer cell line that expresses a high level of hEphA2. In conclusion, the highly specific monobody E1 is useful as a hEphA2 probe candidate for *in vivo* diagnosis and therapy.

## Introduction

Various engineered scaffold proteins to bind specific targets have been studied for *in vitro* research, diagnosis and therapy for human diseases [1]. Monobodies, scaffold proteins originating from the tenth human fibronectin type III domain (Fn3), are one of such proteins that can bind target proteins with high affinity and specificity [2, 3]. Monobodies have advantages for human trials such as a small size for tissue penetration (10 kDa), molecular stability with high melting temperatures (82°C), efficient bacterial production, and an expected low immunity as a protein of human origin [2]. Fn3 has a well-defined structure in which three solvent-accessible loops (BC, DE, and FG) are responsible for binding [4, 5]. To date, various monobody proteins have been developed and tested for clinical efficacy against cancer and infections [3].

Eph receptors and their ligands, ephrins, are important mediators of cell-cell communication and regulate cell attachment to the extracellular matrix, cell shape, and motility [6, 7]. They are associated with tumor progression because high expression of Eph receptors and ephrins correlates with a poor prognosis and high vascularity in cancer tissues. EphA2 is a member of the Eph receptor tyrosine kinase family and is implicated in carcinogenesis including transformation, cell migration, and blood vessel formation [8]. In various cancer types, including melanoma, prostate, breast, colon, lung, pancreatic, and lung cancers, human EphA2 (hEphA2) is highly expressed [8–12]. Ectopic overexpression of hEphA2 provides untransformed epithelial cells with both tumorigenic and metastatic potential [13]. Notably, hEphA2 is present in tumor cells and in the tumor vasculature but not in normal vasculature [14]. The phosphorylation status of hEphA2 after ligand binding also correlates with its oncogenic role because inhibition of hEphA2 receptor activation through its various ligands resulted in decreased phosphorylation concurrent with decreased tumor volume [15, 16].

A number of hEphA2-targeting agents have been developed. Several agonistic monoclonal antibodies, a soluble recombinant ligand ephrin-A1 Fc and small peptides have been shown to be specifically bound with hEphA2 and its overexpressing cells, and to decrease the level of tumor growth and metastasis in mouse models [16–22]. Furthermore, the conjugated drugs also have been studied. A bispecific single-chain antibody against hEphA2 and CD3 has also been shown to effectively promote destruction of hEphA2-expressing tumor cells [23]. Ephrin-A1 conjugated to gold-coated silica nanoshells or *Pseudomonas aeruginosa* exotoxin A has been shown to kill hEphA2-expressing cancer cells in culture [24, 25]. A hEphA2-specific antibody conjugated to a derivative of auristatin, a drug that disrupts microtubules, dramatically inhibits tumor growth in animal models [26, 27]. The 12-mer peptides, designated YSA and SWL, selectively bind to the ephrin-binding domain of hEphA2 and also inhibit ephrin binding to hEphA2 [16, 22]. Furthermore, magnetic nanoparticles and siRNA-loaded nanogels conjugated with YSA have been useful for targeting and removal of cancer cells in the cells and patients [28–31]. Finally, hEphA2-specific antibodies and peptide coupled to imaging agents have been successfully used for tumor visualization in mouse xenograft models [32, 33]. This could be useful for cancer diagnosis, particularly because hEphA2 appears to be overexpressed starting from early stages of cancer [14].

In this study, we developed monobodies that specifically bind hEphA2 from a yeast surface display library, which were then applied to detect tumors in a mouse xenograft model.

## Materials and Methods

### Cell lines and reagents

The extracellular recombinant proteins of hEphA2 and other type-A and type-B Eph (EphA and EphB) homologs were purchased from R&D Systems, MN. To biotinylate hEphA2, lyophilized protein was resuspended in dimethyl sulfoxide (DMSO) and reacted for 1 hr with ten equivalents of biotin-*N*-hydroxysulfosuccinimide (NHS)-ester (Thermo Fisher Scientific, PA) with 2% triethylamine. *E*. *coli* [DH5α and BL21(DE3)], yeast strains (EBY100) and culture media were purchased and used as described [34]. The *Saccharomyces cerevisiae* EBY100 strain was purchased from Invitrogen, NY, and used in all yeast experiments in this work. Selective SD-CAA media for yeast contained 20 g/L glucose, 6.7 g/L yeast nitrogen base without amino acids, and 5.4 g/L Bacto casamino acids. SG-CAA media was identical except glucose was replaced with galactose. The human cell lines, PC3 (prostate) and SKBR3 (breast), were obtained from the American Type Culture Collection (Manassas, VA) and cultured in F-12K (GIBCO, Grand Island, NY) or McCoy's 5A (GIBCO, Carlsbad, CA) media supplemented with 10% fetal bovine serum, respectively. Phosphate-buffered saline (PBS) was from Invitrogen and PBSA was PBS supplemented with 0.1% bovine serum albumin (BSA). All other chemicals were purchased from Thermo Fisher Scientific unless otherwise specified. Various antibodies used in this study were purchased from the indicated companies.

### hEphA2-binding monobody screening with a yeast surface display library

A yeast G4 surface display library (2.5 × 10^8^ diversity), in which EBY100 yeast cells were transformed with pCT surface display vector containing the Fn3 gene with variations of the three binding loop sequences (BC, DE, and FG), was screened with a combination of magnetic bead sorting and fluorescence-activated cell sorting (FACS) with recombinant hEphA2 as described [35]. A single round of yeast isolation consisted of twice isolations with magnetic beads conjugated to hEphA2 and once FACS isolation against double-positive yeasts with 100nM hEphA2 and c-Myc. Then, Fn3 gene fragments were obtained after error-prone polymerase chain reaction (ePCR) against plasmids isolated from sorted yeasts and new libraries for the next rounds of yeast isolation were constructed by transformation of the fragments and pCT vector, as described [34, 36]. Four rounds of yeast isolation were performed to complete the screening. After the fourth round of yeast isolation as described above, yeasts were further isolated after double staining with lower concentration of hEphA2 and an anti-cMyc antibody using a FACSAria III system (BD Biosciences, CA). For FACS isolation, yeasts were stained with 100 nM hEphA2 at room temperature for 2 hr. After a simple wash with PBS containing 0.1% BSA (PBSA), they were incubated with mouse anti-hEphA2 (1:80 dilution, R&D Systems, MN) and chicken anti-cMyc (1:80 dilution, Invitrogen, CA) antibodies at 4°C for 1 hr. Yeasts were then stained with Alexa 488-conjugated goat anti-mouse IgG Fab (1:4000, dilution, Invitrogen, CA) and Alexa 555-conjugated goat anti-chicken IgY (1:80 dilution, Invitrogen, CA) at 4°C for 30 min. Yeasts of the highest hEphA2 region in double-positive fractions were isolated with a FACSAria III system. Yeasts were cultured in 5 mL of SD-CAA media at 30°C and 250 rpm for 1 day. Yeast cultures were centrifuged at 5000 rpm for 1 min and placed into SG-CAA media. At final fourth round, the FACS isolations were repeated twice after staining with 10 nM and 1 nM hEphA2. The final yeast isolate was termed the 4.5 fraction.

Plasmids from the 4.5 fraction were isolated with a Zymoprep Yeast Plasmid Miniprep II kit (ZYMO Research, CA) and amplified in *E*. *coli* DH5a. Ten plasmids isolated from independently transformed *E*. *coli* colonies were sequenced and classified into two groups (E1 and E10) according to their expected monobody amino acid sequence. Two representative plasmids, pCT-Fn3-EphA2(E1) and pCT-Fn3-EphA2(E10) containing the E1 and E10 monobody genes, respectively, were chosen and transformed in EBY100 yeast. The yeasts transformed with each plasmid showed hEphA2 binding similar to that of the 4.5 fraction yeasts isolated with hEphA2.

### Affinity measurements of monobodies against hEphA2 in yeasts

The dissociation constant (Kd) of the monobodies were measured using yeast as described [34, 36]. Yeast cells (2 × 10^6^) transformed with pCT plasmids containing monobody genes were stained with 0.03 to 100 nM hEphA2 overnight at room temperature. Cells were stained with the antibody combinations of described above. After FACS analysis, mean fluorescence values for Alexa 488 and Alexa 555 in double-positive populations of each yeast sample were obtained. Kd values were analyzed by determining the mean[A488]/mean[A555] versus the used hEphA2 concentration using Prism 5 software (Graphpad, CA).

### Monobody purification in *E*. *coli*

To construct expression vectors for monobodies, the genes were amplified with 94oldF (5’-TACATATGGCTAGCGTTTCTGATGTTCCGAG) and 94oldR (5'-TACTGAGTGGATCCTGTTCGGTAATTAATGGAAATTGG) primers against pCT-Fn3-EphA2(E1) and pCT-Fn3-EphA2(E10). The amplified PCR fragments were digested with *Nhe*I and *Bam*HI and ligated into the same sites in the *E*. *coli* pET expression vectors, pETh [37] or pETamh, which contain the same sequence but with the His6 tag (h, HHHHHH) replaced or an AviTag-cMyc-His6 tag (amh, GLNDIFEAQKIEWHEEQKLISEEDLRSHHHHHH) in the C-terminus, respectively. Therefore, E1h and E1amh refer to the E1 monobody with C-terminal h or amh tags, respectively.

Monobody purification was performed in *E*. *coli* BL21(DE3) transformed with bacterial expression vectors as described [37]. Briefly, a single bacterial colony grown on an LB plate supplemented with kanamycin (50 μg/mL) was inoculated into 5 mL of LB media supplemented with kanamycin. After overnight culture, bacteria were transferred to 1 L of LB media and grown at 37°C and 250 rpm for 3 hr. Bacteria were further cultured at 37°C and 250 rpm for 1–3 hr after 1 mL of 0.5 M isopropyl β-D-1-thiogalactopyranoside (IPTG) was added. The bacterial pellet obtained via centrifugation at 3,200 x *g* for 10 min was resuspended in 3 mL of ice-cold lysis buffer [50 mM NaPO_4_ (pH 8.0), 0.5 M NaCl, 5% glycerol, 5 mM CHAPS, 25 mM imidazole, and one complete EDTA-free protease inhibitor cocktail tablet (Roche Applied Science, IN) per 50 mL] and disrupted four times by sonication at 60 W. The supernatants obtained by centrifugation at 12,000 x *g* for 5 min were applied to a HisTrap FF column (GE Healthcare Biosciences, PA) in an AKTA FPLC system (GE Healthcare Biosciences), and His6-tagged proteins were isolated. After acidification with trifluoroacetic acid, proteins were purified again via reverse phase chromatography with a C4 semi-preparative column and then lyophilized. The concentration of purified monobody protein was measured by UV spectrometry [38] after dissolving in DMSO or PBS. Monobody proteins were then stored at—20°C.

### FACS analysis with monobodies

Target-coated magnetic beads were prepared as described [34], bound to monobodies, and analyzed with FACS. Briefly, 6.7 pmol of biotinylated hEphA2 was incubated with 10 μL of streptavidin-coated magnetic beads (Dynabeads Biotin Binder, Invitrogen, CA) in 100 μL of PBSA for 30 min at room temperature. As controls, beads coated with the same amount of biotinylated human IgG (Invitrogen, CA) or lysozyme (Sigma, MO) were prepared. After a simple wash with 1 mL of PBSA, beads were incubated with 0.4 μL of 1 μM monobody in 40 μL of PBSA for 1 hr at room temperature. The monobody proteins bound to beads were stained with 40 μL of a fluorescein isothiocyanate (FITC)-conjugated anti-his tag antibody (1:100 dilution, Abcam, MA) and analyzed by FACS.

Cells were stained with a Cy5.5-labeled monobody (E1h-Cy5.5) and analyzed with FACS. E1h in 0.1 M sodium bicarbonate buffer (pH 9.5) was chemically conjugated with Cy5.5-NHS (BioActs, Korea) in DMSO with a 1:2 molar ratio at 4°C for 16 hr. After removing unreacted Cy5.5 with a PD-10 desalting column (GE Healthcare, NJ), proteins were further purified with an analytical high performance liquid chromatography system using C18 analytical columns (Grace/Vydac, Fisher Scientific, CA) in 20–80% gradient of solvent B for 30 min. Gradient was made of solvent A (0.1% trifluoroacetic acid in distilled water) and solvent B (0.1% trifluoroacetic acid in acetonitrile). Cells (0.5×10^5^) were incubated with 100 nM E1h-Cy5.5 in 100 μL of PBSA for 2 hr at room temperature. As a control, unstained cells were measured.

### Determination of total and surface hEphA2 levels in cells

The relative amount of total hEphA2 protein was measured by western blotting. Cell pellets (1×10^6^ cells) detached from cell culture plates were resuspended in SDS sample buffer and boiled for 5 min. After separation by 10% SDS-PAGE, proteins were transferred onto a nitrocellulose membrane (Bio-Rad, Hercules, CA), and hEphA2 was detected with a mouse anti-hEphA2 antibody (1:500 dilution, Santa Cruz Biotechnology, CA) and horseradish peroxidase-conjugated goat anti-mouse IgG (1:2000 dilution, Lifetechnology, Inc., Grand Island, NY) combination. As a loading control, beta-actin was detected using the same (stripped) membrane. Immunoreactive proteins were detected using luminal reagent (Santa Cruz Biotechnology, CA) and visualized by a Fuji Film image reader LAS-3000 machine.

The amount of hEphA2 on the cell surface was quantitated with FACS and compared to that of an anti-rat antibody-coated bead standard (Bangs Laboratories, IN). Cells (2.5×10^5^) were stained with 100 μL of mouse anti-hEphA2 antibody (1:100 dilution, R&D Systems, MN) for 30 min on ice. After washing, the cells were resuspended in 50 μL of PBSA, mixed with 50 μL of FITC-conjugated rat anti-mouse IgG (1:50 dilution, BioLegend, CA), and incubated on ice for 30 min. At the same time, one drop (50 μL) of each Bangs bead was mixed with 50 μL of FITC-conjugated rat anti-mouse IgG and incubated on ice for 30 min. After washing, mean fluorescence values for each cell and Bangs bead were measured by FACS. A standard curve was generated using the mean values of each Bangs bead versus the antibody concentration, and EphA2 concentration on the cell surface was calculated.

### Enzyme-linked immunosorbant assay (ELISA) against EphA receptors

ELISA plates (Costar, Sigma) were coated with 100 μL of a 1 μg/mL solution of each recombinant EphA receptors and incubated overnight at 4°C. On the next day, the plates were washed three times with PBS containing 0.05% Tween 20 (PBST) and blocked with 300 μL of blocking buffer (PBS containing 5% BSA) for 2 hr at room temperature. 100 μL of E1amh was added to each well and incubated at room temperature for 2 hr. Plates were washed, and 100 μL of horseradish peroxidase-conjugated mouse anti-cMyc antibody (1:1,000 dilution, Invitrogen, CA) was added for 30 min at room temperature. After washing and drying, plates were incubated with 100 μL of a substrate (1:1 mix of a 3,3',5,5'-tetramethylbenzidine substrate and hydrogen peroxide, Thermo Fisher Scientific, NH) for 5 min at room temperature. The reaction was stopped with 50 μL of 1 M H_2_SO_4_, and the ELISA signal was read at 450 nm.

### Animal tumor model preparation and optical imaging by E1 monobody

Male BALB/c athymic nu-/nu- mice (5–6 weeks old) were purchased from the Orient Company, Korea. Mice subcutaneously transplanted with PC3 cells were prepared as described [39]. Animal care, all experiments, and the euthanasia procedures were performed in accordance with protocols approved by the Chonnam National University Animal Research Committee (Permit Number: CNU IACUC-H-2014-1). Anesthesia was performed using 2% isoflurane for injection of cells, E1h-Cy5.5 monobodies, and imaging. After transplantation of PC3 cells (1×10^8^ cells in 100 μL of PBS), tumors were grown for 21–28 days to a size of 150 mm^3^. Nude mice transplanted with PC3 cells were intravenously injected with non-labeled E1h (30 μg) or PBS. At day 1, E1h-Cy5.5 (6 μg) was intravenously injected into the same mice. optical images of the tumors were obtained with an IVIS 100 system (Caliper, MA) at day 6. After imaging, we observed the fluorescence remaining in the animal after the organs and tumor tissue were removed. Specifically, tumor tissues were sliced and observed via fluorescence microscopy.

### Cytotoxicity and *in vivo* stability of E1h

The cytotoxicity of E1h was determined by 3-(3,4-dimethylthiazol-2-yl)-2,5-diphenyltetrazolium (MTT) assay as described [40]. Cells (10,000 per well) cultured for 1 day in 46-well plates were replaced with medium containing 100 nM E1h after washed with PBS, and further cultured. The indicated time point later, the surviving cells were stained with MTT and quantified by absorbance at 540 nm. The MTT assay results were plotted with mean ± S.D. of three experiments.

The *in vivo* stability of E1h was measured by western blotting against E1h in tumor tissue obtained from PC3-bearing mice after *in vivo* optical imaging. The tumor tissues dissected from mice were soaked in PBS buffer containing 1X protease inhibitor cocktail solution (GenDEPOT) and grinded with homogenizer. The tumor samples were centrifuged in 12,000 rpm for 10 min. The supernatants (100 μg protein) were mixed with SDS sample buffer and separated with 12% SDS-PAGE. After transferred to nitrocellulose membranes, proteins were first probed using a mouse anti-his tag antibody (1:1000 dilution, Santa Cruz Biotechnology, CA) and then a horseradish peroxidase-conjugated anti rabbit secondary antibody (1:2000 dilution, Amersham, UK). As a loading control, beta-actin was detected using the same (stripped) membrane.

### Statistical analysis

Statistical analysis was performed using the SPSS 21.0 statistical packages (SPSS, Chicago, IL). Statistical analysis was performed using the two-tailed Student’s *t* test or two-way ANOVA. A *P* value of <0.05 was considered statistically significant (*P<0.05, **P < 0.01). All data are expressed as means ± SD.

## Results

### Screening of hEphA2-binding monobodies via a yeast surface display library

**Fig 1:**
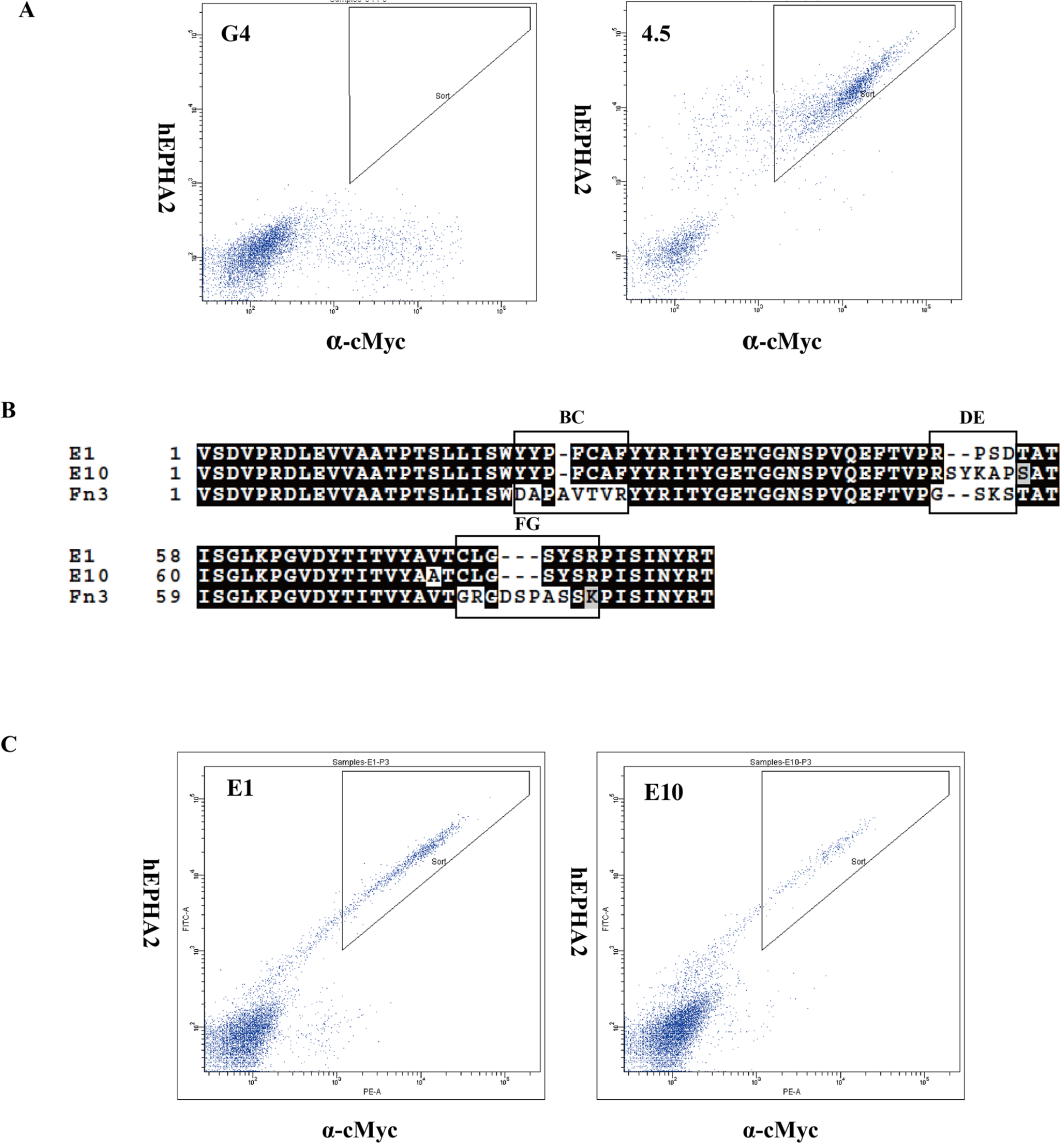
Screening of EphA2-binding monobodies in a G4 yeast surface display library. (A) FACS profiles of the original G4 library (left) and 4.5-fraction yeasts (right) obtained after final screening. Yeast cells were stained with 1 nM hEphA2 and a chicken anti-cMyc antibody. (B) Amino acid sequence alignment of monobodies and wild-type Fn3 domain. Sequences of the BC, DE, and FG loops are indicated as black boxes. (C) FACS profiles of yeasts transformed with surface display vectors, pCT-Fn3-EphA2 (E1) and pCT-Fn3-EphA2 (E10). Yeast cells were stained with 10nM hEphA2 and chicken anti-cMyc antibody.

To obtain a hEphA2-binding monobody, we screened an yeast G4 library. The final isolated yeast fraction (fraction 4.5) showed higher affinity for hEphA2 than those obtained from the original yeast library (Fig 1A). In *E*. *coli* transformed with pCT plasmids obtained from fraction 4.5 yeasts, ten independent clones were randomly chosen. Their plasmids were sequenced and classified into two groups: E1 and E10. Nine plasmids were classified into the E1 group and one into the E10 group. Some plasmids included in the E1 group exhibited one or two mutations in the backbone amino acid sequence. We chose two plasmids, pCT-EphA2(E1) and pCT-EphA2(E10), because their monobody genes did not show any mutations in the backbone (Fig 1B). They carried the same sequences except in the DE loop. Their amino acid sequences and loop lengths were completely different from that of wild-type Fn3. We concluded that they are hEphA2-binding monobodies because we observed that yeasts transformed with each plasmid showed hEphA2 binding in their FACS profiles (Fig 1C).

### Affinity measurements of monobodies against hEphA2

**Fig 2:**
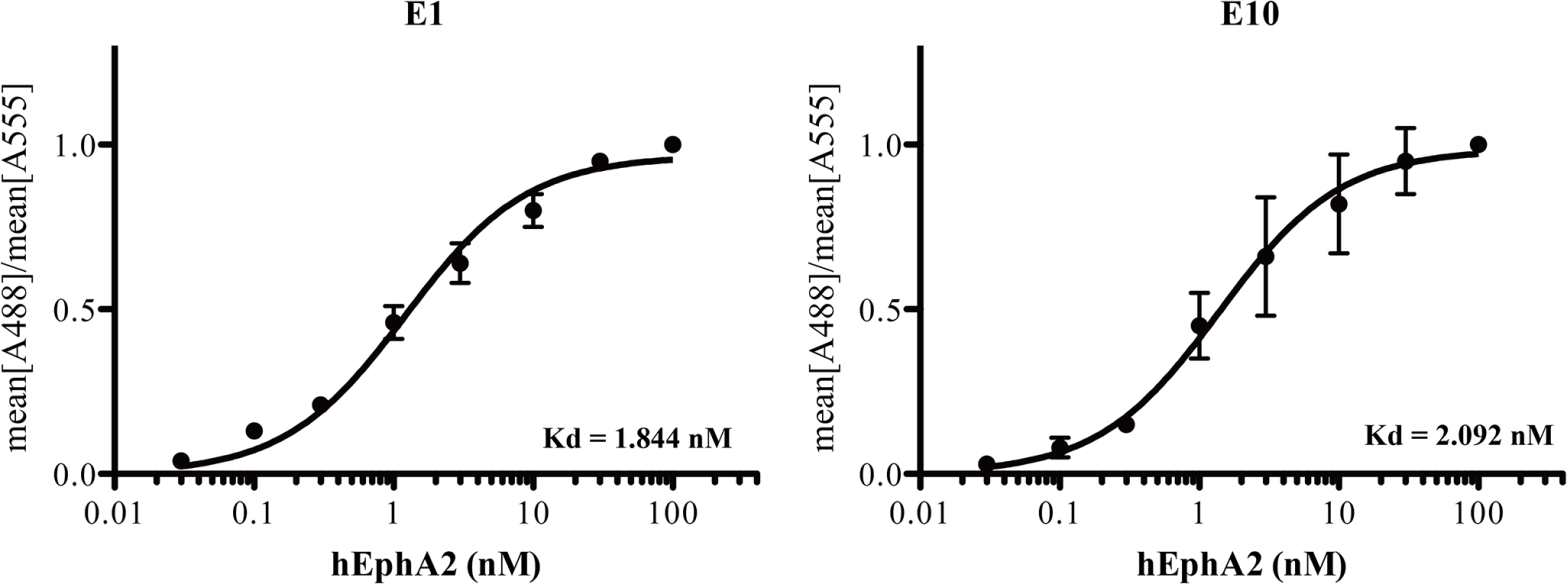
Affinity measurements of E1 and E10 monobodies against hEphA2. The indicated concentrations of hEphA2 protein were incubated with yeasts expressing each monobody. After a simple wash, yeast cells were stained with mouse anti-hEphA2 and chicken anti-cMyc antibodies, followed by staining with Alexa 488-conjugated anti-mouse and Alexa 555-conjugated anti-chicken secondary antibodies. The mean fluorescence values of each sample were measured by FACS. Affinities (Kd values) were obtained by determining the mean ratio of Alexa 488/Alexa 555 fluorescence versus hEphA2 concentration. The results are representatives of at least three independent experiments.

To measure the affinity of each monobody against target proteins, we incubated yeasts transformed with monobody-carrying plasmids with various concentrations of hEphA2 overnight at room temperature. The amount of hEphA2 bound to yeast cells was measured by FACS analyses (Fig 2). The Kd values were ~2 nM (1.844 nM for E1 and 2.092 nM for E10). These values were comparable to the affinities of other monobody proteins [3] and typical antibodies [41].

### Target specificity of hEphA2-binding monobodies

**Fig 3:**
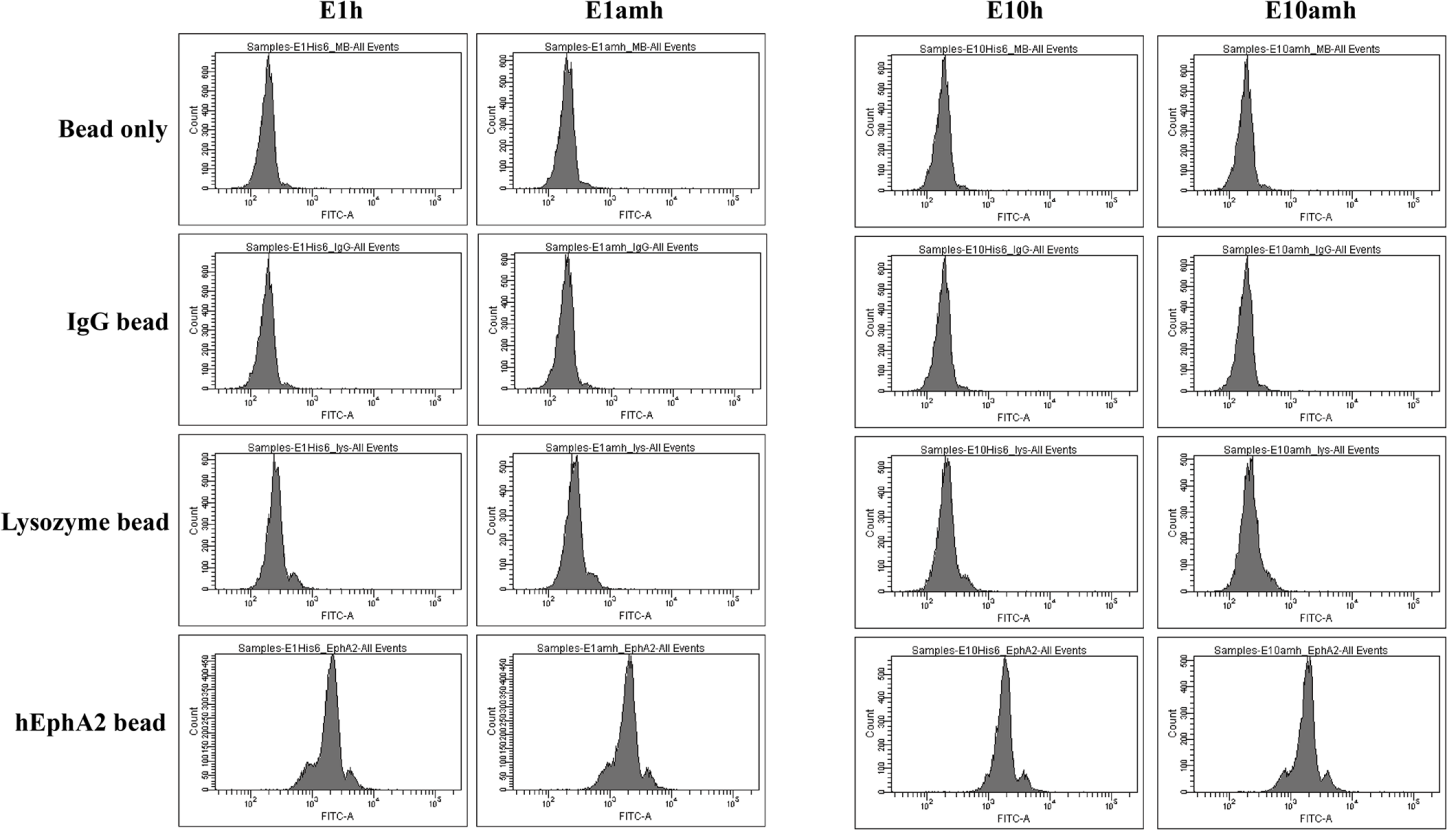
Binding of monobodies against target-bound beads. Streptavidin-coated magnetic beads bound with biotinylated human IgG, lysozyme, or hEphA2 were incubated with 10 nM monobody proteins with His6 (h) or AMH tags at room temperature. The bound monobodies were stained with a FITC-conjugated anti-His-tag antibody and analyzed by FACS. The results are representatives of at least three independent experiments.

We purified the recombinant h- or amh-tagged E1 and E10 monobodies in *E*. *coli* BL21(DE3) using affinity chromatography. First, we measured binding to beads coated with various proteins by FACS analysis (Fig 3). Beads coated with human IgG or lysozyme showed mean fluorescence levels similar to those of the beads alone after incubation with E1 and E10 proteins. By contrast, hEphA2-coated beads showed much higher levels of binding to these proteins in their FACS profiles.

**Fig 4:**
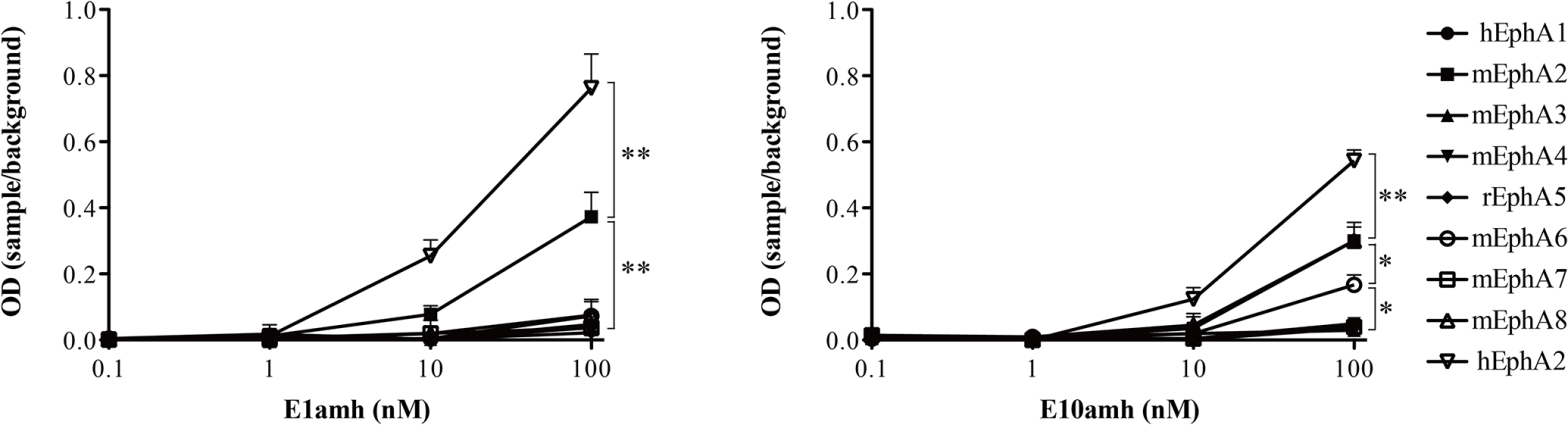
Binding of monobody proteins to recombinant type-A Eph receptors. The indicated concentrations of AMH-tagged monobody proteins were incubated for 2 hr on 96-well plates coated with recombinant Eph receptor proteins. The amounts of bound monobody proteins were measured in an ELISA reader after staining with horseradish peroxidase-conjugated anti-cMyc antibodies. Data represent mean ± S.D., and asterisks (*) indicate a significant difference compared hEphA2 and other EphA series (*, P < 0.05 or **, P < 0.01). The results are representatives of at least three independent experiments.

Next, we performed ELISA analyses with hEphA2 and its homologs (Fig 4) because hEphA2 has a 90% amino acid sequence homology to mEphA2 but 25–35% homologies with other Eph receptors [6]. hEphA1 is the most homologus gene to hEphA2 in them [42]. Various concentrations of amh-tagged monobody proteins were incubated on 96-well plates coated with various EphA homolog proteins. E1amh and E10amh proteins were highly bound to hEphA2 and moderately bound to mEphA2. At 100 nM monobody concentration, E1amh did not show significant binding to other homologs. However, E10amh proteins were significantly bound to mEphA8 and mEphA6. These results indicated that E1 only bound to hEphA2 and mEphA2 receptors.

### Binding of the E1 monobody against hEphA2 expressed in cells and in a tumor model mouse

**Fig 5:**
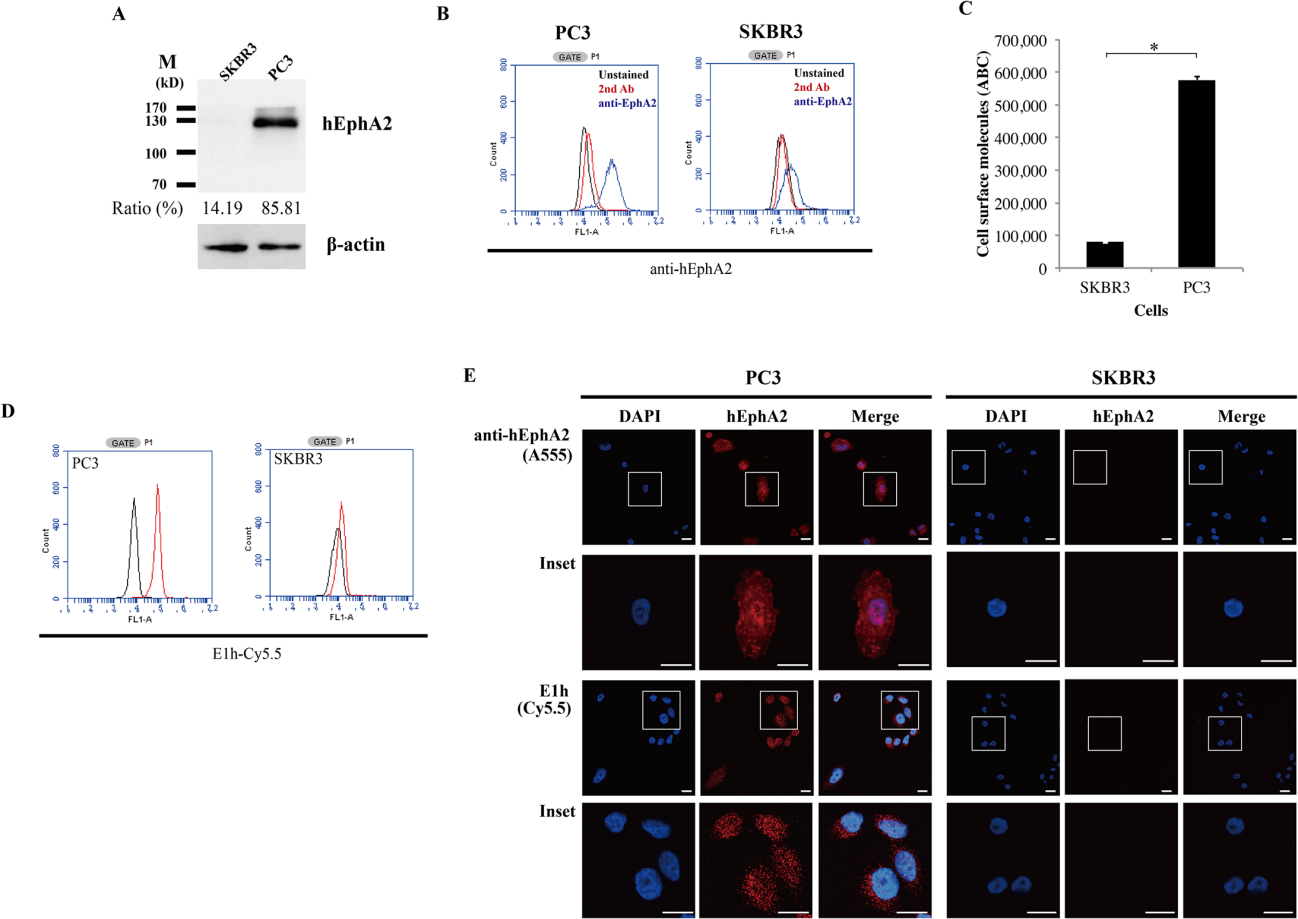
Binding of monobody proteins to hEphA2-expressing cells. (A) Western blotting for hEphA2. Cell lysates were separated by 10% SDS-PAGE and transferred onto nitrocellulose membranes. The proteins were detected with an anti-hEphA2 antibody. (B) Surface expression of hEphA2. Cells (2 × 10^5^) were stained with a mouse anti-hEphA2 antibody and FITC-conjugated rat anti-mouse IgG antibody. Black line, unstained cells; red line, only second Ab; blue line, anti-EphA2. (C) Quantitation of hEphA2 on the cell surface. Concentrations of hEphA2 on the cell surface were measured against an anti-rat Bangs bead standard. Data represent mean ± S.D., and asterisks (*) indicate a significant difference compared SKBR3 and PC3 cells (*, P < 0.001). (D) Measurement of E1 monobody binding to cells. Cells were incubated with 100 nM Cy5.5-conjugated E1h monobody and analyzed by FACS. Black lines, unstained cells; red lines, E1h-Cy5.5. (F) Fluorescence microscopy images of cells stained with an anti-hEphA2 antibody or E1h-Cy5.5 monobody. Cell nuclei were stained with 4',6-diamidino-2-phenylindole (DAPI). Magnification of the inset with indivisual and merged images is given in the lower low. Scale bar, 20 μm. The results are representatives of at least three independent experiments.

**Fig 6:**
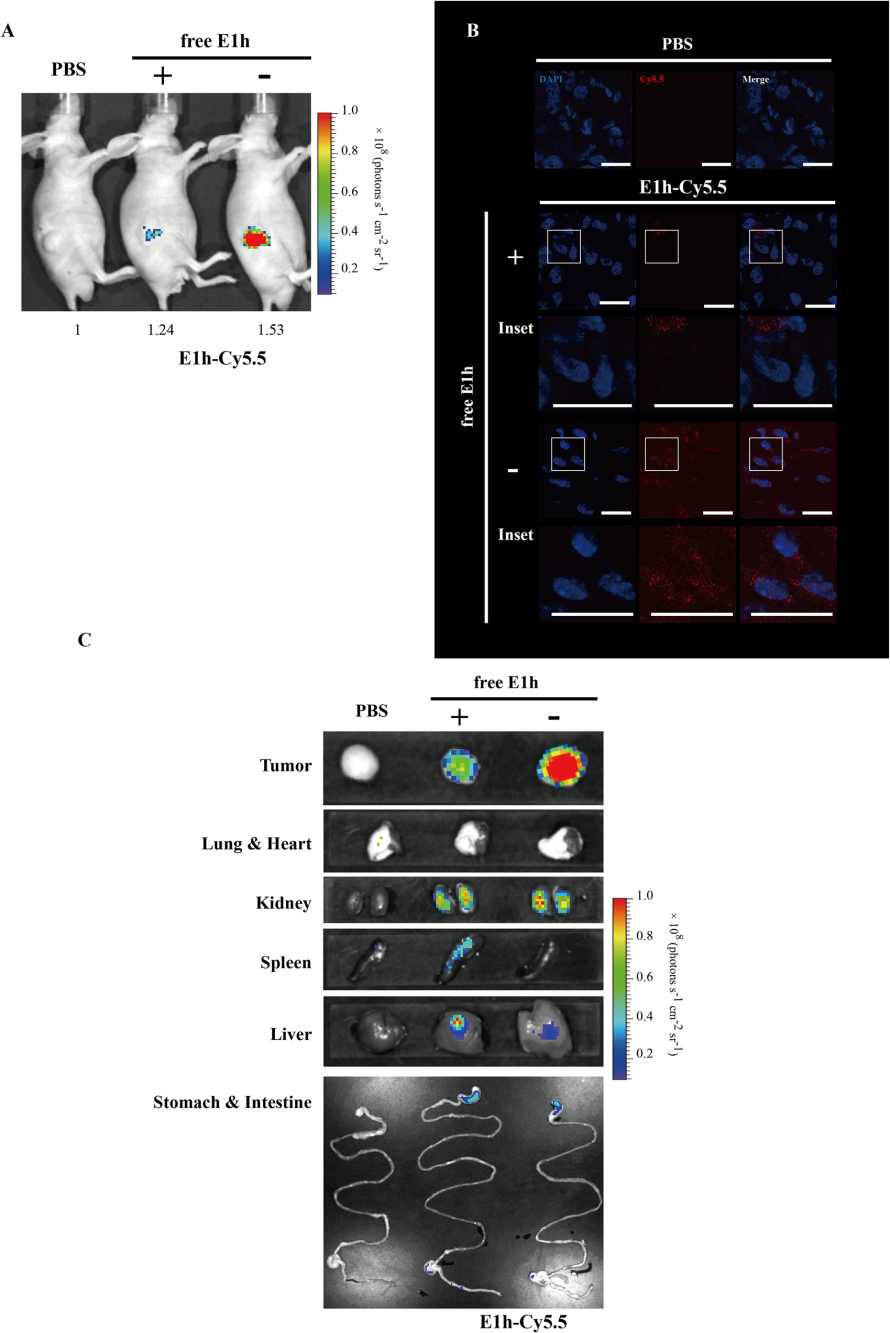
*In vivo* targeting of the E1h monobody. BALB/c athymic nu^-^/nu^-^ were injected subcutaneously with PC3 (1 × 10^7^). When the tumors reached a volume of approximately 150 mm^3^, PC3 tumor bearing mice were treated with PBS (n = 4), or E1h-Cy5.5 (n = 8). (A) Optical images of PC3 tumor tissues after E1h-Cy5.5 injection into mice. Nude mice were injected with non-tagged E1h (30 μg, n = 4) (+) or without (-) (n = 4). After 1 day, E1h-Cy5.5 (6 μg) was injected into the same mice. At day 6, fluorescence images of the tumors were obtained with an IVIS 100 system. Data for each tumor are normalized to the average radiance of the PBS group. (B) Fluorescence microscopy images of PC3 tumor tissues. Tumor tissues dissected in (A) were sliced and the fluorescences by Cy5.5 were observed. For clear visualization by confocal microscopy, the cell nuclei (blue) were stained with DAPI, whereas Cy5.5 is shown in red. Magnification of the inset with indivisual and merged images is given in the lower low. Scale bar, 20 μm. (C) The optical images of the indicated organs and tissues from (A). They were obtained with an IVIS system. The results are representatives of at least three independent experiments.

Finally, we assessed whether E1 monobody could be used as a probe for detecting hEphA2 expressed in cells and tissues. To do this, we conjugated E1h with fluorescent dye Cy5.5 and named E1h-Cy5.5 (Figs 5 and 6).

It was previously reported that hEphA2 is expressed in PC3 (prostate cancer) and SKBR3 (breast cancer) cell lines at high and low levels, respectively [11, 26]. The same results were also obtained in our analysis (Fig 5). PC3 cells expressed hEphA2 at 6-fold higher levels than SKBR3 cells, as shown by western blotting and FACS analyses using an anti-hEphA2 antibody (Fig 5A, 5B and 5C). Both cell lines were incubated with 100 nM E1h-Cy5.5 and analyzed by FACS (Fig 5D). PC3 cells treated with E1h-Cy5.5 showed a 20-fold increase in mean fluorescence intensity compared to that of non-treated cells. However, SKBR3 cells showed little change in their FACS profile. Similarly, fluorescence microscopy images obtained with the anti-hEphA2 antibody or E1h-Cy5.5 were similar to each other in PC3 cells, whereas fluorescence signals were not observed in SKBR3 cells (Fig 5E).

We tested whether the E1 monobody specifically targeted and bound hEphA2 expressed in animal models (Fig 6 and S2 Fig). We transplanted PC3 cells highly expressing hEphA2 into nude mice in skin to induce human tumor xenografts. To test for specific targeting, non-labeled E1h (30 μg per mice) was injected via the tail vein at day 0. At next day, E1h-Cy5.5 was injected via the tail vein (6 μg per mice) and the optical imaging intensity in mice was obtained at day 6 (Fig 6A and S2 Fig). The optical intensity in mouse tumor tissue after treatment of non-labeled E1h was less obtained than that without the treatment (1.24 after treatment and 1.53 without treatment). This indicated that E1h-Cy5.5 specifically binds hEphA2 in PC3 tumor tissues and that pretreatment with non-labelled E1h inhibits E1h-Cy5.5 binding in tumor tissue. The E1h-Cy5.5 binding decrease by the presence of non-tagged E1h was similar in fluoroscopy images of tumor tissues dissected from transplanted mice (Fig 6B). We also measured optical imaging intensity in the organs obtained from such mice. The levels were much lower than those of tumor tissue (Fig 6C).

## Discussion

Here, we report two novel monobodies, E1 and E10, which specifically bind hEphA2,. In a sequence analysis, we found that the two monobodies had the same amino acid sequences except in the DE loop in which E1 and E10 contained 4 and 6 amino acids, respectively. Such a loop length is acceptable in the Fn3 scaffold backbone structure and can be used to design a library [43]. However, we found that only one E10 clone was among ten clones randomly chosen from the final isolated yeast fractions.

The DE loop sequence is more important for target specificity, but not binding, than the other two loops. The Kd values of the E1 and E10 monobodies were not significantly different but E10 showed significant binding to other EphA homologs such as mEphA6 and mEphA8 as well as hEphA2 in the ELISA analysis following 100 nM monobody treatment. Our monobody sequences did not carry an RGD motif in the FG loop. This is a binding motif for integrin, an original target of the Fn3 protein [44]. This shows that the monobody sequences were isolated during the library screening steps.

Most antibodies have Kd values in the low micromolar (10^−6^) to nanomolar (10^−7^ to 10^−9^) range. High affinity antibodies are generally considered to be in the low nanomolar range (10^−9^) [34]. Our monobodies showed Kd values of below 2 nM, which should be of sufficient affinity to use as a candidate for *in vitro* and *in vivo* applications. Here, we showed that E1 targeted cells and tissue expressing hEphA2. With 1 nM E1h, hEphA2 could be detected in the FACS analysis (Figs 1 and 3). However, E1h itself is not cytotoxic because the viability of PC3 and SKBR3 cells was not affected by treatment with 100 nM E1h for 5 days as well as immunoblot analysis of excised tumor tissue demonstrated that E1h was maintained in tumors as described IVIS imaging (S1A and S1B Fig). To develop E1 as a tumor therapy, another methodology will be required.

Currently, specific drugs targeting hEphA2 are being developed. A human monoclonal antibody (1C1) specifically bound to hEphA2 with a Kd value of 0.4 nM, and when conjugated to a cytotoxic drug, effectively inhibited xenograft tumors in mice [26, 32]. Peptides such as SWL and YSA have also been studied as probes [28]. A number of monobodies are being tested in preclinical or clinical trials [3, 45]. Thus we hope that our E1 monobody can be developed to novel hEphA2-specific drugs.

## Supporting Information

S1 Fig *In vitro* cytotoxicity and *in vivo* stability of E1h. (A) *In vitro* cytotoxicity against tumor cells. SKBR3 and PC3 cells were *in vitro* cultured and incubated with E1h (100 nM) for five days. Cell viability after E1h treatment was measured by MTT assay at the indicate time points. (B) *In vivo* stability of E1h. Immunoblot analysis against E1h exsited in PC3 tumor tissue of the nude mice in Fig 6 after injected by PBS, free E1h with (+) or without (-) E1h-Cy5.5. The result is one of representatives of three independent experiments.
(TIF)

S2 Fig Supplementary *in vivo* targeting of the E1h monobody. BALB/c athymic nu^-^/nu^-^ with subcutaneous PC3 tumor cells were generated and treated with PBS (n = 4) and E1h (n = 8) via tail vein as described in Materials and mothods. One day later, the mice treated with E1h were re-treated with (+) or without (-) E1h-Cy5.5 and At day 6, the fluorescence images of the mice and tumors were obtained with an IVIS 100 system. The images from mice and tumors were except those in Fig 6.
(TIF)
