# Supplementary figures and images for: Isolation and Characterization of a Monobody with a Fibronectin Domain III Scaffold That Specifically Binds EphA2
Source: PLoS One. 2015 Jul 15;10(7):e0132976. doi: 10.1371/journal.pone.0132976 (PMC4503726; doi:10.1371/journal.pone.0132976)

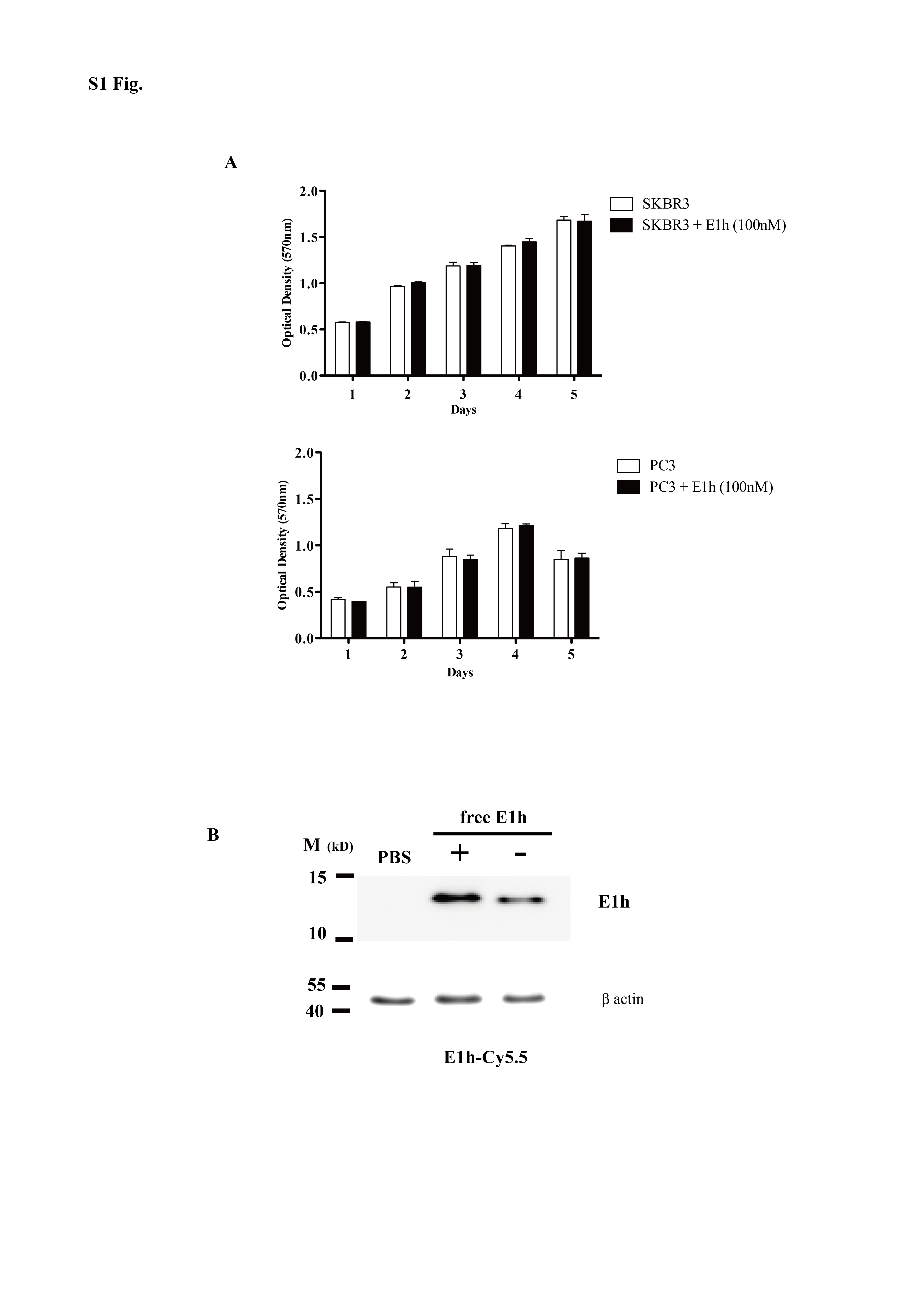

Supplement: S1 Fig — (A) In vitro cytotoxicity against tumor cells. SKBR3 and PC3 cells were in vitro cultured and incubated with E1h (100 nM) for five days. Cell viability after E1h treatment was measured by MTT assay at the indicate time points. (B) In vivo stability of E1h. Immunoblot analysis against E1h exsited in PC3 tumor tissue of the nude mice in Fig 6 after injected by PBS, free E1h with (+) or without (-) E1h-Cy5.5. The result is one of representatives of three independent experiments. (TIF) [file pone.0132976.s001.tif]

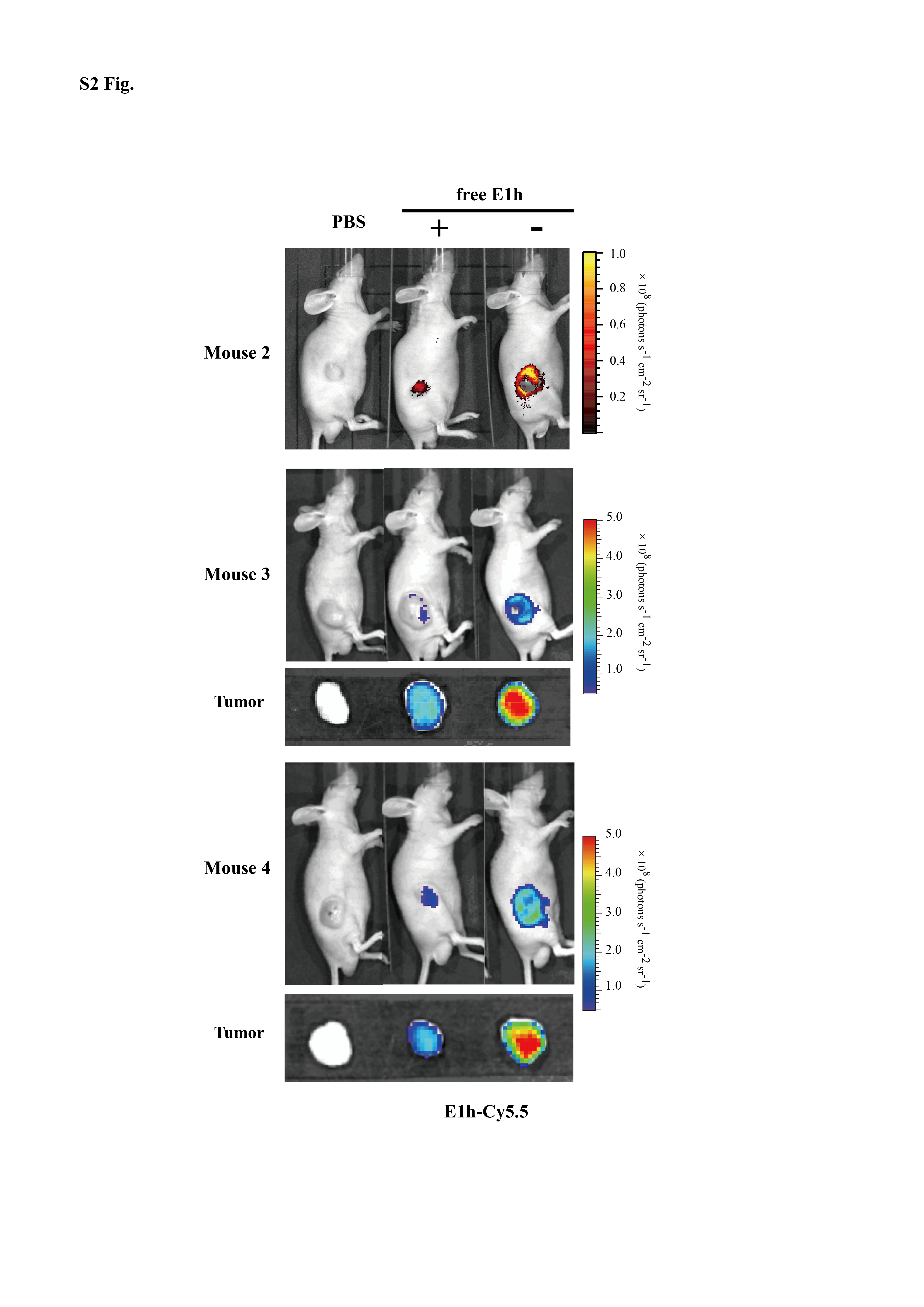

Supplement: S2 Fig — BALB/c athymic nu-/nu- with subcutaneous PC3 tumor cells were generated and treated with PBS (n = 4) and E1h (n = 8) via tail vein as described in Materials and mothods. One day later, the mice treated with E1h were re-treated with (+) or without (-) E1h-Cy5.5 and At day 6, the fluorescence images of the mice and tumors were obtained with an IVIS 100 system. The images from mice and tumors were except those in Fig 6. (TIF) [file pone.0132976.s002.tif]
